# Comparison of contemporary transcatheter heart valve prostheses: data from the German Aortic Valve Registry (GARY)

**DOI:** 10.1007/s00392-023-02242-z

**Published:** 2023-07-18

**Authors:** Tanja K. Rudolph, Eva Herrmann, Dimitria Bon, Thomas Walther, Timm Bauer, Stephan Ensminger, Christian Frerker, Andreas Beckmann, Helge Möllmann, Raffi Bekeredjian, Friedhelm Beyersdorf, Christian Hamm, Stephan Baldus, Andreas Böning, Jan Gummert, Volker Rudolph, Sabine Bleiziffer

**Affiliations:** 1 Clinic for General and Interventional Cardiology/Angiology, Herz- und Diabeteszentrum NRW, Ruhr-Universität Bochum, Georgstr. 11, 32545 Bad Oeynhausen, Germany; 2 Institute of Biostatistics and Mathematical Modelling, Goethe University Frankfurt, Frankfurt am Main, Germany; 3 German Center for Cardiovascular Research, DZHK, Partner Site Rhein-Main, Bad Nauheim, Germany; 4 Department of Cardiac Surgery, Goethe University Hospital, Frankfurt, Germany; 5 Department of Cardiology, Intensive Care and General Internal Medicine, Sana Klinikum Offenbach, Offenbach, Germany; 6 Department of Thoracic and Cardiovascular Surgery, University Hospital, Lübeck, Germany; 7 Department of Cardiology, Faculty of Medicine and University Hospital Cologne, University of Cologne, Cologne, Germany; 8 German Society for Thoracic and Cardiovascular Surgery, Langenbeck-Virchow-Haus, Berlin, Germany; 9 Medizinische Klinik I, St.-Johannes-Hospital Dortmund, Dortmund, Germany; 10 Department of Cardiology, Robert-Bosch Hospital, Stuttgart, Germany; 11 Department of Cardiovascular Surgery, University Heart Center, Freiburg, Germany; 12 Department of Cardiology, Kerckhoff Clinic, Bad Nauheim, Germany; 13 Department of Cardiovascular Surgery, University Hospital Giessen, Giessen, Germany; 14 Clinic for Thoracic and Cardiovascular Surgery, Herz- und Diabeteszentrum NRW, Ruhr-Universität Bochum, Bad Oeynhausen, Germany

**Keywords:** Aortic stenosis, Transcatheter valve prosthesis, All-comers data, Head-to-head comparison

## Abstract

**Background:**

Various second-generation transcatheter heart valve (THV) prostheses with high clinical efficacy and safety are available, but there is limited large-scale data available comparing their hemodynamic performance and clinical implications.

**Objective:**

To compare the hemodynamic performance and short-term clinical outcome of four second-generation THV prostheses.

**Methods:**

24,124 patients out of the German Aortic Valve Registry who underwent transfemoral transcatheter aortic valve implantation (TAVI) (Evolut™ R *n* = 7028, Acurate neo™ *n* = 2922, Portico *n* = 878 and Sapien 3 *n* = 13,296) were included in this analysis. Propensity-score weighted analysis was performed to control for differences in age, left ventricular function, STS score and sex. Primary endpoint was survival at one-year, secondary endpoints were 30 days survival, pre-discharge transvalvular gradients, paravalvular leakage and peri-procedural complications.

**Results:**

Thirty-day and one-year survival were not significantly different between the four patient groups. Transvalvular gradients in Evolut™ R and Acurate neo™ were significantly lower as compared to Portico and Sapien 3 at hospital discharge. This difference exists across all annulus sizes. Paravalvular leakage ≥ II occurred significantly less often in the Sapien 3 group (1.2%, *p* < 0.0001). Rate of severe procedural complications was low and comparable in all groups. Permanent pacemaker implantation rate at one year was lowest in the ACUARATE neo group (13.0%) and highest in the Evolut™ R group (21.9%).

**Conclusion:**

Albeit comparable short-term clinical outcomes there are certain differences regarding hemodynamic performance and permanent pacemaker implantation rate between currently available THV prostheses which should be considered for individual prosthesis selection.

**Graphical Abstract:**

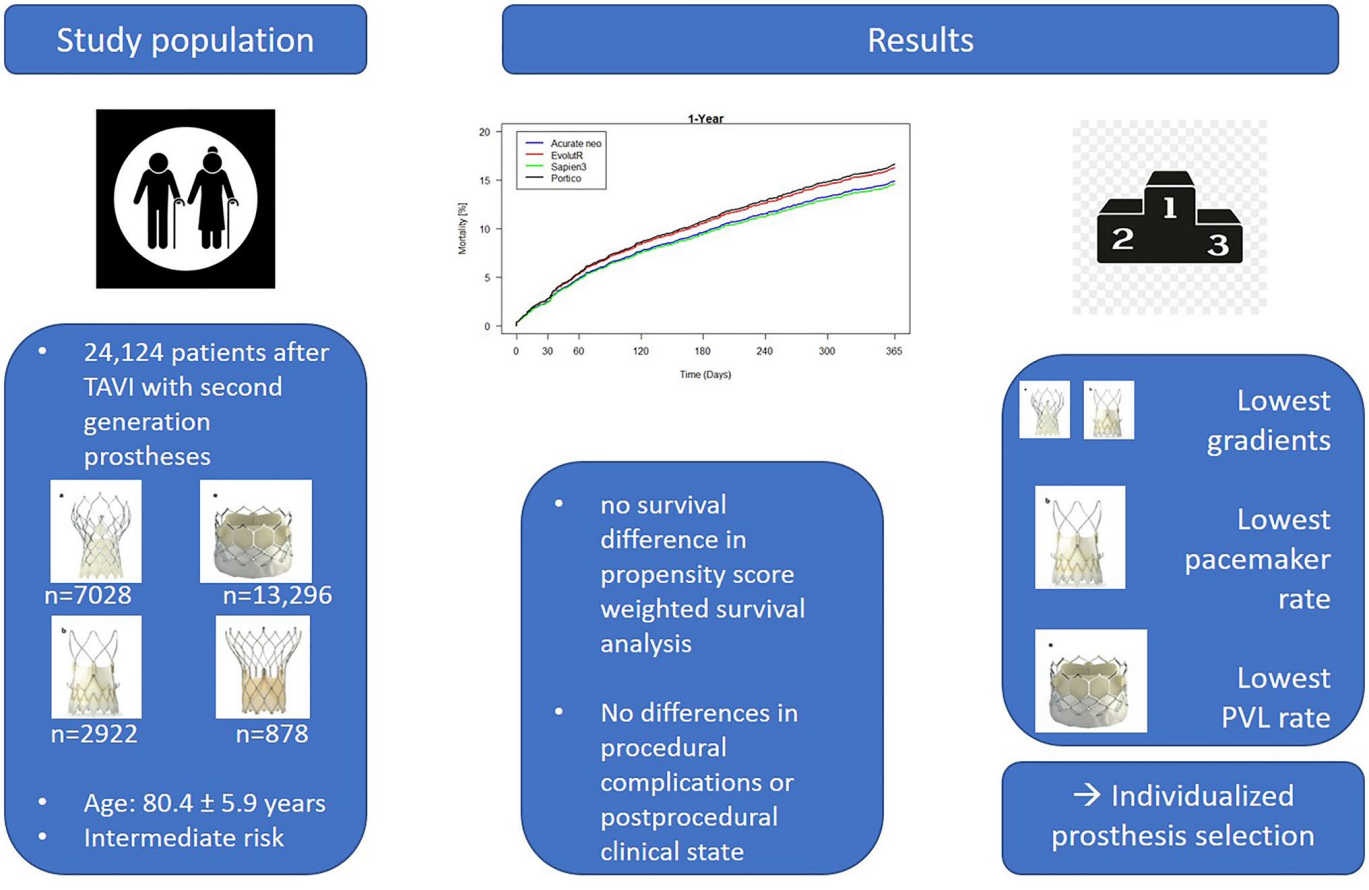

**Supplementary Information:**

The online version contains supplementary material available at 10.1007/s00392-023-02242-z.

## Introduction

Transcatheter aortic valve implantation (TAVI) has become a safe and standardized procedure. With the development of second-generation transcatheter heart valve (THV) prostheses significantly lower rates of procedure-related complications and higher clinical success could be observed after TAVI as compared to first-generation THV prostheses. One balloon-expandable THV prosthesis (Sapien 3) and three self-expanding THV prostheses (Acurate neo™, Evolut™ R and Portico) are frequently used. Due to the different prosthesis design (intra-annular versus supra-annular) and distinct implantation mode a difference in hemodynamic performance, clinical outcome as well as safety profile could be hypothesized.

Previous studies suggest there might be differences in transvalvular gradients, paravalvular leakage rates, permanent pacemaker implantation, and cerebrovascular event rates [1–8]. However, CE mark studies and post-market registries of these four prostheses showed an excellent safety profile [4, 9–11]. Clinical outcomes as well as 30-day and 1-year mortality seem to be similar for the four THV prostheses, however, there is limited direct large-scale comparison, in particular in all-comers populations.

To fill this gap of knowledge we analyzed the data of all patients included in the German Aortic Valve Registry (GARY) who were treated with a transfemoral TAVI with either the balloon-expandable Sapien 3 or the self-expanding Evolut^TM^R, Acurate neo™ or Portico THV prosthesis.

## Methods

Data of all patients who underwent transfemoral TAVI between 2014 and 2019 were extracted from the database of GARY which is a nationwide multicenter all-comers registry. The registry design has been previously published [12]. Prostheses studied in this analysis included the balloon-expandable SAPIEN 3 (Edwards Lifesciences, Irvine, CA, USA) and the self-expanding Acurate neo™ (Boston Scientific, Marlborough, MA, USA), Evolut™ R (Medtronic, Minneapolis, MN, USA) and Portico (Abbott). Prostheses selection was at the discretion of the operating physician.

Transvalvular gradients and paravalvular leakage were assessed pre-discharge by transthoracic echocardiography. Procedural data, procedural success and severe intraprocedural complications were analyzed.

Patients were followed-up at 30 days and one year regarding adverse clinical events and NYHA classification by phone interviews. One-year follow-up was not available in patients undergoing transfemoral TAVI in 2016 and 2017. The primary outcome was the mortality rate at one year.

### Ethical statement

The study complies with the Declaration of Helsinki. Subjects included in the registry gave informed consent, and an ethics body at participating institutions approved the use of patient data for research purposes.

### Statistics

Continuous variables are reported as mean ± standard deviation (SD) for the total patient cohort or mean ± standard error (SE) for group comparisons. Categorical variables are reported as frequencies and percentages. Comparisons between the different prosthesis groups were made using unweighted and weighted linear and generalized linear models. For adjusted comparisons with weighted regression analysis, a propensity score model from boosted logistic regression analysis was used to determine weights to estimate the average treatment effect of the group receiving the Acurate neo™ devices as a reference. Variables included in the propensity score model were age, gender, LVEF (≤ 30%, 31–50%,  > 50%) and STS score. Adjusted analysis according to this propensity score model was also used to compare 30-day and one-year mortality in the patient groups with a weighted Cox proportional hazard model.

Tests with a two-sided *p*-value of ≤ 0.05 were considered statistically significant. Statistical analysis was performed with R (R Foundation for Statistical Computing, Vienna, Austria). The packages “twang”, “gbm” and “survey” were used for calculating propensity score weights.

## Results

### Baseline characteristics

Overall, 24,124 patients who underwent transfemoral TAVI have been included in this retrospective analysis. Mean age ± SD was 80.4 ± 5.9 years and there was a female predominance, in particular in self-expanding prostheses (Table 1). The percentage of comorbidities is summarized in Table 1. Occurrence of peripheral and coronary artery disease was significantly different between the four groups as well as left ventricular function. Patients treated with Sapien 3 showed a more severe aortic valve calcification (Supplementary Table 1).

**Table 1:** Baseline characteristics

	All (*n* = 24,124)	ACURATE neo (*n* = 2922)	Portico (*n* = 878)	Evolut R (*n* = 7028)	Sapien 3 (*n* = 13,296)	*p* value
Age (years), mean ± SD/SE^1,2^	80.4 ± 5.9	80.3 ± 0.11	80.9 ± 0.20	80.3 ± 0.07	80.5 ± 0.05	0.038
Weighted comparison		80.3 ± 0.07	80.4 ± 0.07	80.3 ± 0.07	80.4 ± 0.07	0.920
Male gender, *n*/*N* (%)^2^	11,630/24,123 (48.2%)	1083/2922 (37.1%)	294/877 (33.5%)	3022/7028 (43.0%)	7231/13296 (54.4%)	< 0.0001
Weighted comparison		37.1%	36.9%	37.0%	37.2%	0.995
BMI (kg/m^2^), mean ± SD/SE^1^	27.6 ± 5.4	28.0 ± 0.10	27.9. ± 0.18	27.5 ± 0.06	27.5 ± 0.05	< 0.0001
		28.0 ± 0.07	28.1 ± 0.07	27.6 ± 0.07	27.7 ± 0.07	< 0.0001
EF (%), mean ± SD/SE^1^	52.4 ± 13.4	54.4 ± 0.24	54.2 ± 0.43	53.0 ± 0.16	51.4 ± 0.12	< 0.0001
LVEF ≤ 30%, *n*/*N* (%)^2^	2012/24,124 (8.3%)	164/2922 (5.6%)	42/878 (4.8%)	518/7028 (7.4%)	1288/13,296 (9.7%)	< 0.0001
Weighted comparison		5.6%	4.9%	5.7%	5.6%	0.487
LVEF ≤ 50, *n*/*N* (%)^2^	8672/24,124 (35.9%)	878/2922 (30.0%)	256/878 (29.2%)	2421/7028 (34.4%)	5117/13,296 (38.5%)	< 0.0001
Weighted comparison		30.0%	29.9%	30.4%	30.3%	0.968
Mean transvalvular gradient (mmHg), mean ± SD/SE^1^	42.2 ± 17.1	41.6 ± 0.31	43.5 ± 0.57	41.6 ± 0.20	42.61 ± 0.15	< 0.0001
Aortic annulus diameter (mm), mean ± SD/SE^1^	24.4 ± 2.9	23.9 ± 0.05	23.8 ± 0.10	24.2 ± 0.03	24.7 ± 0.03	< 0.0001
STS-Score, mean ± SD/SE^1,2^	5.58 ± 4.41	5.23 ± 0.082	5.33 ± 0.149	5.82 ± 0.053	5.55 ± 0.038	< 0.0001
Weighted comparison		5.23 ± 0.048	5.20 ± 0.048	5.22 ± 0.048	5.19 ± 0.048	0.929
CAD, *n*/*N* (%)	13,481/24,124 (55.9%)	1476/2922 (50.5%)	452/878 (51.5%)	3895/7028 (55.4%)	7658/13,296 (57.6%)	< 0.0001
Diabetes, *n*/*N* (%)	7902/24,114 (32.8%)	1013/2922 (34.7%)	312/877 (35.6%)	2270/7027 (32.3%)	4307/13,288 (32.4%)	0.026
PAD, *n*/*N* (%)	6327/24,105 (26.2%)	698/2921 (23.9%)	159/876 (18.2%)	1867/7026 (26.6%)	3603/13,282 (27.1%)	< 0.0001
COPD, *n*/*N* (%)	3849/24,095 (16.0%)	468/2920 (16.0%)	125/877 (14.3%)	1127/7020 (16.1%)	2129/13,278 (16.0%)	0.558
Neurological dysfunction, *n*/*N* (%)	3675/24,100 (15.2%)	432/2920 (14.8%)	129/877 (14.7%)	1078/7026 (15.3%)	2036/13,277 (15.3%)	0.851
Hypertension, *n*/*N* (%)^2^	21,177/23,726 (89.3%)	2706/2919 (92.7%)	772/846 (91.3%)	6264/7006 (89.4%)	11,435/12955 (88.3%)	< 0.0001
Weighted comparison		92.7%	91.2%	89.1%	88.5%	< 0.0001
Renal insufficiency, *n*/*N* (%)^2^	12,842/24,124 (53.2%)	1363/2922 (46.6%)	425/878 (48.4%)	3785/7028 (53.9%)	7269/13,296 (54.7%)	< 0.0001
Weighted comparison		46.6%	49.6%	49.6%	49.2%	< 0.0001
Frailty, *n*/*N* (%)^2^	12,767/24,124 (52.9%)	1983/2922 (67.9%)	496/878 (56.5%)	3861/7028 (54.9%)	6427/13,296 (48.3%)	< 0.0001
Weighted comparison		67.9%	56.6%	55.8%	49.3%	< 0.0001
Previous surgery, *n*/*N* (%)^2^	3935/24,110 (16.3%)	366/2922 (12.5%)	116/877 (13.2%)	1391/7027 (19.8%)	2062/13,284 (15.5%)	< 0.0001
Weighted comparison		12.5%	14.1%	17.0%	12.8%	< 0.0001

^1^Data are given as mean ± standard deviation (SD) in the group of all patients and mean ± standard error (SE) for group comparisons in the other columns. Shown are standard comparisons as well as weighted analysis results

^2^Variables used for propensity score adjustments

*p* values represent a global comparison between prothesisi groups using propensity scoe adjustments

Only Evolut™ R and Sapien 3 valves are available for annulus sizes > 27 mm, thus mean annulus sizes are significantly larger in these groups (24.2 mm and 24.7 mm for Evolut™ R and Sapien 3, versus 23.9 mm and 23.8 mm for Acurate neo™ and Portico, *p* < 0.001, Table 1). To account for systematic data differences, weighted analysis with weights from a propensity score model using adjustments for age, gender, STS score and LV function was also performed. See Table 1 and Supplemental Fig. 1 for the adjustment effects.

### Clinical outcome

**Fig. 1:**
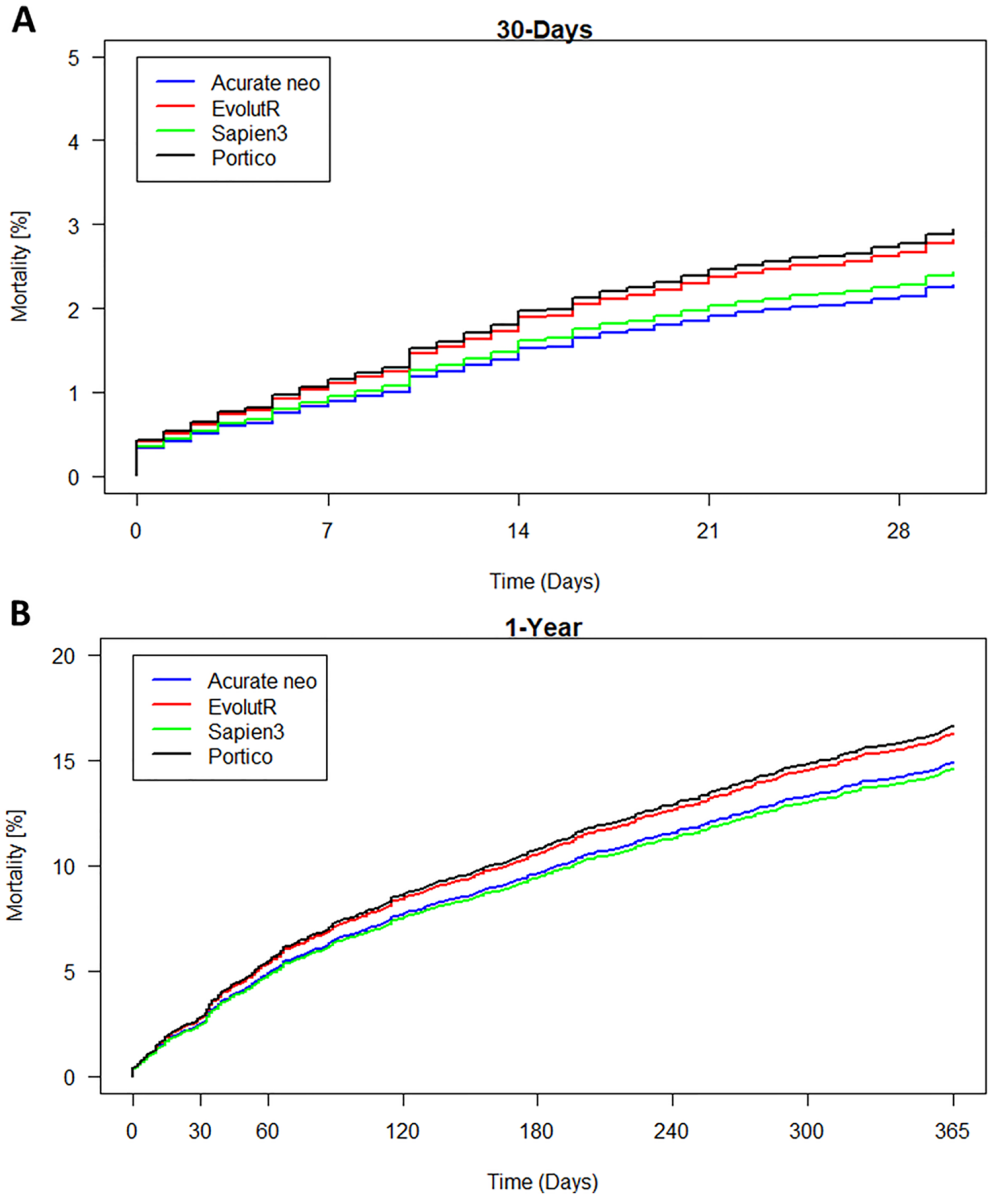
Mortality after 30 days (**A**) and one year (**B**) with propensity score derived weights adjusting for age, gender, left ventricular ejection fraction and STS score. Mortality was comparable for all studied TAVI prostheses with no significant differences even in not significance-adjusted pairwise comparisons

Overall survival in the study cohort was 97.2% (95% CI 97.0–97.4%) at 30 days and 83.0% (95% CI 82.3–83.7%) at 1 year, respectively. There was no significant difference between the four groups in the propensity score-adjusted analysis (Fig. 1A and B). To explore the effect of aortic annulus size this parameter was also included as a covariate. Furthermore, as sensitivity analysis, we restricted the data to a subset with an aortic annulus diameter between 21 and 27 mm (*n* = 19,504, Supplemental Fig. 2). Both approaches yielded no significant differences between the four groups.

**Fig. 2:**
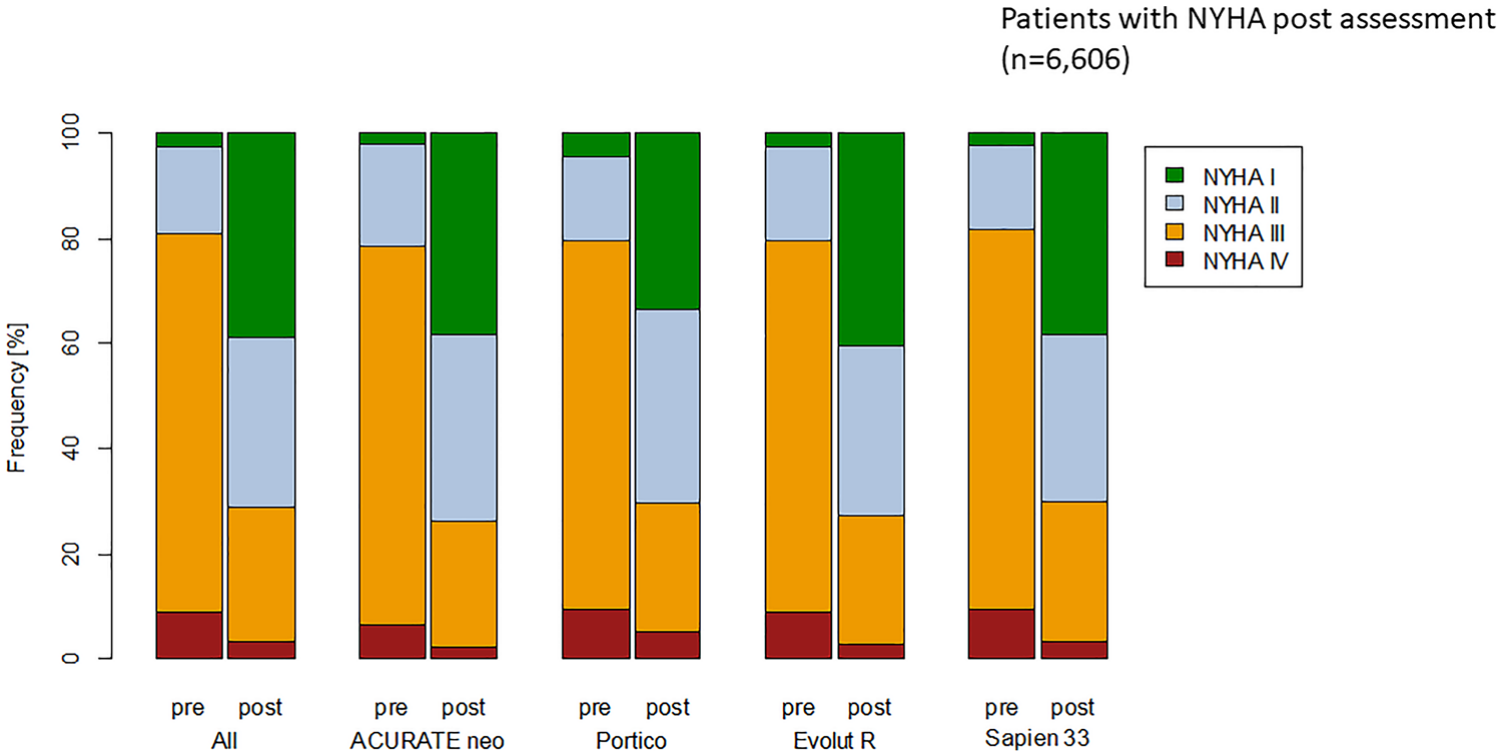
NYHA class at baseline (pre) and after 1 year (post) showing a clear improvement following TAVI without any significant difference between the four groups. Results are shown for all 6.606 patients with 1 year follow-up assessment. Supplemental Fig. 2 shows NYHA class at baseline in all 24,124 patients

Pre-procedurally, patients were symptomatic with dyspnoea mainly in NYHA class III and IV and improved significantly after 1 year, regardless of the implanted TAVI prosthesis (Fig. 2 for unadjusted comparisons, results were even more comparable between the groups after weighting). In adjusted comparisons, there was no significant difference between the groups regarding stroke rate, TIA rate in patients without stroke, rehospitalization, reintervention and myocardial infarction.

Permanent pacemaker implantation rate at 1 year was lowest in the Acurate neo™ group (13.0%) and highest in the Evolut™ R group (23.0%, Table 2, *p* < 0.0001). Results for adjusted weighted comparisons were similar (Table 2).

**Table 2:** Clinical endpoints per patient at one year follow-up

	All (*n* = 7487)	Acurate neo (*n* = 841)	Portico (*n* = 206)	Evolut R (*n* = 1713)	Sapien 3 (*n* = 4727)	*p* value
Myocardial Infarction, *n*/*N* (%)	45/6765 (0.7%)	10/768 (1.3%)	1/192 (0.5%)	12/1547 (0.8%)	22/4258 (0.5%)	0.135
Propensity weighted comparison		1.3%	0.6%	0.6%	0.5%	0.253
Stroke, *n*/*N* (%)	110/6777 (1.6%)	11/766 (1.4%)	3/192 (1.6%)	23/1546 (1.5%)	73/4273 (1.7%)	0.907
Propensity weighted comparison		1.4%	1.5%	1.4%	1.5%	0.997
TIA but no Stroke *n*/*N* (%)	120/6760 (1.8%)	11/767 (1.4%)	3/192 (1.6%)	34/1544 (2.2%)	72/4257 (1.7%)	0.518
Propensity weighted comparison		1.4%	1.6%	2.0%	1.8%	0.857
New pacemaker/ICD, *n*/*N* (%)	1142/6076 (18.8%)	92/707 (13.0%)	36/166 (21.7%)	322/1399 (23.0%)	692/3804 (18.2%)	< 0.0001
Propensity weighted comparison		13.0%	21.6%	21.9%	16.5%	< 0.0001
PCI, *n*/*N* (%)	57/6751 (0.8%)	10/766 (1.3%)	1/191 (0.5%)	8/1543 (0.5%)	38/4251 (0.9%)	0.228
Propensity weighted comparison		1.3%	0.6%	0.6%	0.8%	0.456
Further Hospitalisation, *n*/*N* (%)	2573/6762 (38.1%)	293/769 (38.1%)	70/190 (36.8%)	585/1545 (37.9%)	1625/4258 (38.2%)	0.983
Propensity weighted comparison		38.1%	37.7%	36.8%	37.1%	0.951
Further Hospitalisation due to complications related to the aortic valve intervention, *n*/*N* (%)	192/6733 (2.9%)	18/766 (2.3%)	5/188 (2.7%)	45/1539 (2.9%)	124/4240 (2.9%)	0.832
Propensity weighted comparison		2.3%	2.3%	2.7%	2.8%	0.913
Further Hospitalisation due to heart or circulatory problems, *n*/*N* (%)	1037/6729 (15.4%)	101/764 (13.2%)	30/189 (15.9%)	242/1537 (15.7%)	664/4239 (15.7%)	0.348
Propensity weighted comparison		13.2%	16.2%	15.4%	14.9%	0.446
Reintervention, *n*/*N* (%)	51/7487 (0.7%)	7/841 (0.8%)	1/206 (0.5%)	9/1713 (0.5%)	34/4727 (0.7%)	0.762
Propensity weighted comparison		0.8%	0.5%	0.5%	0.6%	0.840

### Hemodynamic performance

Pre-procedural mean transvalvular gradient was comparable between the four groups (Table 1), whereas post-procedurally mean transvalvular gradient was significantly lower in the Acurate neo™ group (mean ± SE 8.60 ± 0.14 mmHg, *p* < 0.0001, Table 3) versus the overall cohort. The differences remained significant in the weighted analysis and in a sensitivity analysis in a subgroup of patients with aortic annulus diameter of < 23 mm, 23–25 mm and > 25 mm. Data on effective orifice areas have not been collected in GARY.

**Table 3:** Hemodynamics at discharge

	All (*n* = 24,124)	ACURATE neo (*n* = 2922)	Portico (*n* = 878)	Evolut R (*n* = 7028)	Sapien 3 (*n* = 13,296)	*p* value
Paravalvular leakage						
None/trace *n*/*N* (%)	15,084/23,652 (63.8%)	1741/29,13 (59.8%)	410/857 (47.8%)	3839/6997 (54.9%)	9094/12,885 (70.6%)	< 0.0001
Propensity weighted comparison		59.8%	48.9%	54.7%	70.6%	< 0.0001
Grade ≥ II *n*/*N* (%)	387/23,652 (1.6%)	60/2913 (2.1%)	26/857 (3.0%)	149/6997 (2.1%)	152/12,885 (1.2%)	< 0.0001
Propensity weighted comparison		2.1%	3.1%	2.0%	1.2%	< 0.0001
Grade ≥ III *n*/*N* (%)	32/23,652 (0.1%)	7/2913 (0.2%)	1/857 (0.1%)	7/6997 (0.1%)	17/12,885 (0.1%)	0.4454
Propensity weighted comparison		0.2%	0.1%	0.1%	0.1%	0.4583
Mean transvalvular gradient (mmHg), mean ± SD/SE^1^	10.45 ± 7.86	8.60 ± 0.14	9.29 ± 0.25	8.82 ± 0.09	11.83 ± 0.07	< 0.0001
Propensity weighted comparison		8.60 ± 0.09	9.27 ± 0.09	8.80 ± 0.09	12.11 ± 0.09	< 0.0001

Subgroup Aortic annulus diameter < 23 mm	All (*n* = 5832)	Acurate neo (*n* = 748)	Portico (*n* = 233)	Evolut R (*n* = 1,900)	Sapien 3 (*n* = 2951)	
Mean transvalvular gradient (mmHg), mean ± SD/SE^1^	11.34 ± 7.83	9.61 ± 0.27	10.27 ± 0.48	9.73 ± 0.17	13.01 ± 0.14	< 0.0001
Propensity weighted comparison		9.61 ± 0.20	10.35 ± 0.20	9.56 ± 0.20	13.15 ± 0.20	< 0.0001

Subgroup Aortic annulus diameter 23–25 mm	All (*n* = 10,736)	Acurate neo (*n* = 1594)	Portico (*n* = 451)	Evolut R (*n* = 3114)	Sapien 3 (*n* = 5577)	
Mean transvalvular gradient (mmHg), mean ± SD/SE^1^	10.23 ± 7.04	8.44 ± 0.17	9.04 ± 0.31	8.54 ± 0.12	11.82 ± 0.09	< 0.0001
Propensity weighted comparison		8.44 ± 0.12	9.01 ± 0.12	8.53 ± 0.13	11.94 ± 0.13	< 0.0001

Subgroup Aortic annulus diameter > 25 mm	All (*n* = 7321)	Acurate neo (*n* = 560)	Portico (*n* = 191)	Evolut R (*n* = 1964)	Sapien 3 (*n* = 4606)	
Mean transvalvular gradient (mmHg), mean ± SD/SE^1^	10.06 ± 8.90	7.71 ± 0.37	8.54 ± 0.66	8.34 ± 0.20	11.12 ± 0.13	< 0.0001
Propensity weighted comparison		7.71 ± 0.18	8.51 ± 0.18	8.39 ± 0.16	11.34 ± 0.16	< 0.0001

^1^Data are given as mean ± standard deviation (SD) in the group of all patients and mean ± standard error (SE) for group comparisons in the other columns. Shown are standard comparisons as well as weighted analysis results

The observed overall rate of clinically relevant paravalvular leakage (grade II +) at discharge was 1.6% ranging from 1.2% in the Sapien 3 group to 2.1% and 3.1% in the Acurate neo™ and Portico groups, respectively (Table 3, *p* < 0.0001). Again, the differences remained similar in extent and significance in the weighted analysis and in the sensitivity subset of patients with aortic annulus diameter of < 23 mm, 23–25 mm and > 25 mm. Only 32 out of 23,652 patients with available data displayed grade III or IV paravalvular leakage.

### Procedural data

Procedural characteristics are listed in Table 4. Radiation time and amount of contrast dye were significantly lower in the ACUARTE neo™ and the Sapien 3 group (Table 4). Pre-dilatation rate was highest in Acurate neo™ and Portico (84.7% and 82.0%) and lowest in Evolut™ R (55.2%). Post-dilatation rate was significantly lower in Sapien 3 (13.1%). Overall procedural success rate was high (98.3%) without significant difference between the groups (Table 4).

**Table 4:** Procedural and postoperative data

	All (*n* = 24,124)	ACURATE neo (*n* = 2922)	Portico (*n* = 878)	Evolut R (*n* = 7,028)	Sapien 3 (*n* = 13,296)	*p* value
Radiation time (min), mean ± SD/SE^1^	14.40 ± 17.42	13.84 ± 0.32	15.17 ± 0.59	16.46 ± 0.21	13.39 ± 0.15	< 0.0001
Propensity weighted comparison		13.84 ± 0.22	15.18 ± 0.22	16.17 ± 0.22	13.22 ± 0.22	< 0.0001
Contrast dye (ml), mean ± SD/SE^1^	132.50 ± 71.01	132.67 ± 1.31	145.82 ± 2.39	140.93 ± 0.84	127.13 ± 0.61	< 0.0001
Propensity weighted comparison		132.67 ± 0.91	145.85 ± 0.92	140.92 ± 0.92	126.04 ± 0.92	< 0.0001
Pre-dilatation, *n*/*N* (%)	15,141/24,124 (62.8%)	2475/2922 (84.7%)	720/878 (82.0%)	3877/7028 (55.2%)	8069/13,296 (60.7%)	< 0.0001
Propensity weighted comparison		84.7%	81.7%	55.9%	60.9%	< 0.0001
Post-dilatation, *n*/*N* (%)	5078/24,124 (21.0%)	1076/2922 (36.8%)	339/878 (38.6%)	1924/7028 (27.4%)	1739/13,296 (13.1%)	< 0.0001
Propensity weighted comparison		36.8%	38.0%	27.7%	13.7%	< 0.0001
Procedural Success, *n*/*N* (%)	21,120/21,493 (98.3%)	2426/2487 (97.5%)	752/768 (97.9%)	5873/5978 (98.2%)	12,069/12,260 (98.4%)	0.0230
Propensity weighted comparison		97.5%	97.8%	98.2%	98.5%	0.0671
Vascular complication during procedure, *n*/*N* (%)	699/24,123 (2.9%)	108/2922 (3.7%)	40/878 (4.6%)	281/7028 (4.0%)	270/13,295 (2.0%)	< 0.0001
Propensity weighted comparison		3.7%	4.6%	3.9%	2.0%	< 0.0001
Arterial vascular complication up to 30 days, *n*/*N* (%)	1769/24,124 (7.3%)	248/2922 (8.5%)	80/878 (9.1%)	511/7028 (7.3%)	930/13,296 (7.0%)	0.0089
Propensity weighted comparison		8.5%	8.9%	7.3%	7.1%	0.0318
Conversion to surgery, *n*/*N* (%)	99/24,124 (0.4%)	12/2922 (0.4%)	6/878 (0.7%)	25/7028 (0.4%)	56/13,296 (0.4%)	0.5917
Propensity weighted comparison		0.4%	0.7%	0.4%	0.4%	0.3610
Coronary obstruction *n*/*N* (%)	36/24,123 (0.1%)	3/2922 (0.1%)	3/878 (0.3%)	8/7028 (0.1%)	22/13,295 (0.2%)	0.4004
Propensity weighted comparison		0.1%	0.4%	0.1%	0.2%	0.0536
Immediate valve-in-valve *n*/*N* (%)	134/21,458 (0.6%)	10/2484 (0.4%)	6/768 (0.8%)	55/5977 (0.9%)	63/12,229 (0.5%)	0.0062
Propensity weighted comparison		0.4%	0.8%	0.9%	0.5%	0.1298
Intraoperative death, *n*/*N* (%)	55/24,124 (0.2%)	2/2922 (0.1%)	4/878 (0.5%)	16/7028 (0.2%)	33/13,296 (0.2%)	0.0981
Propensity weighted comparison		0.1%	0.4%	0.2%	0.2%	0.0335
Intra- or postprocedural stroke *n*/*N* (%)	272/24,019 (1.1%)	29/2911 (1.0%)	13/874 (1.5%)	84/6994 (1.2%)	146/13,240 (1.1%)	0.6167
Propensity weighted comparison		1.0%	1.5%	1.2%	1.1%	0.4195
Intra- or postprocedural myocardinfarct *n*/*N* (%)	79/24,124 (0.3%)	10/2922 (0.3%)	3/878 (0.3%)	20/7028 (0.3%)	46/13,296 (0.3%)	0.9019
Propensity weighted comparison		0.3%	0.4%	0.3%	0.3%	0.9441

^1^Quantitative data are given as mean ± standard deviation (SD) in the group of all patients and mean ± standard error (SE) for group comparisons in the other columns. Shown are standard comparisons as well as weighted analysis results

The rate of vascular complications during the procedure was 2.9% with a significantly lower rate in the Sapien 3 group (2.0%, *p* = 0.003) which was also confirmed in adjusted analysis using propensity score weights. Severe intraprocedural complications occurred very infrequently (Table 4). Conversion rate to open heart surgery was reported in 0.4% of patients without differences between the groups. Coronary obstruction was reported in 36 out of 24,124 patients (0.1%). An immediate valve-in-valve implantation was performed in 134 out of 21,458 patients (0.6%) without any difference between the four groups.

## Discussion

This study comprises a large-scale multi-center comparison of hemodynamic performance and short-term clinical outcome of the four most implanted second-generation THV prostheses. The main findings in this all-comers cohort are (1) one-year survival rates are not different between the various second-generation THV prostheses; (2) short-term outcome and hemodynamic performance are excellent for all THV prostheses with some certain differences of lower transvalvular gradients in supra-annular self-expanding valves and less paravalvular leakage in balloon-expandable valves; (3) a high procedural success rate combined with a low rate of severe intraprocedural complications, which confirms excellent efficacy and safety of all studied devices.

### Clinical outcome

30-day and one-year survival rates were high in all studied second-generation THV prostheses (97.2% and 83.0%, respectively) in this intermediate-risk cohort and are in line with data from previous publications [4, 5, 7, 9–11, 13–15]. In contrast to one recent study from the French TAVI registry there was no difference in survival between balloon- and self-expanding THV prostheses [16] This might be explained by the fact that in the French registry patients received only first-generation self-expanding THVs which are more prone to a higher rate of clinically significant paravalvular regurgitation, periprocedural complications including stroke and permanent pacemaker implantation [17].

Short- and intermediate-term clinical event rates were low which underlines the high safety of all four THV prostheses.

Rehospitalisation within a year was reported in approximately 40% of the patients in the current analysis mainly due to cardiovascular reasons in about 15%. There was a significant improvement in clinical symptoms which is comparable to previous studies in an intermediate-risk cohort [18].

The stroke rate at one year was only 1.6%, which is significantly lower as compared to previous publications [7, 13, 15, 17, 19–21]. This might in part be due to underreporting in the registry-based data acquisition.

The rate of new permanent pacemaker implantation (PPI) was 18.8% after one year in the overall cohort. Differences between different devices have previously been described [4, 6, 22]. However, the rate is significantly higher as compared to randomized studies in low- and intermediate-risk patients [2, 3, 22, 23]. As reported before [15], Acurate neo™ displayed the lowest rate of PPI among self-expanding vales (13.0%) which might be due to the lower radial force of the stent as well as to the different implantation mode with a top-down deployment resulting in lower mechanical stress on the left ventricular outflow tract and subsequentially on the AV conduction system.

### Procedural data and hemodynamics

The incidence of severe intraoperative complications was extremely confirming a high safety for all TAVI platforms investigated. Radiation time and usage of contrast medium was significantly lower in Acurate neo™ and Sapien 3 which is mainly due to the distinct implantation modes.

Pre-dilatation rate was highest in the Acurate neo™ and Portico group since the official recommendation is to pre-dilate the calcified native valve before implantation At the time of data acquisition Sapien 3 was also mainly implanted with pre-dilatation, however, current data have shown that Sapien 3 can safely be implanted without pre-dilation [24–26]. Post-dilatation rate was significantly lower in the balloon-expandable Sapien 3 [4, 10–12] and most probably a result of the high radial force of this valve [27]. Pre- and post-ballooning did not impact stroke rate in this cohort.

Overall peri interventional vascular access complication rate was in line with other reports in intermediate-risk patients [4, 12]. Notably, vascular complication rate was lowest in balloon-expandable THV which might be partially explained by the lower proportion of female patients who are prone to access site complications.

Baseline transvalvular gradients were comparable and there was a significant reduction in all four groups. Of interest, transvalvular gradients were lowest in the self-expanding Evolut™ R and Acurate neo™ even after adjusting for various annulus sizes. This finding is mainly explained by the supra-annular valve design which translates into lower transvalvular gradients. Among the intra-annular prosthesis types, the self-expanding Portico shows lower gradients compared to the balloon-expandable Sapien 3. Previous publications have reported the same finding and, furthermore, showed a significantly higher effective orifice area and a lower rate of patient prosthesis mismatch in supra-annular valves [2, 3]. There is evidence that patient prosthesis mismatch is a strong predictor for cardiac remodelling, development of heart failure and even premature prosthesis degeneration [28]. Since effective orifice area has not been collected in GARY we are not able to make any statements regarding this issue from our data, but at least the results of the transvalvular gradients emphasize that there is a difference in hemodynamics between the studied THV devices which might impact long-term outcome.

Paravalvular leakage is a major limitation of TAVI and has been linked to reduced survival in various studies [16, 22]. As opposed to first-generation THV devices all studied THV showed an acceptably low rate of clinically relevant paravalvular leakage being lowest with the balloon-expandable Sapien 3 device (1.2% PVL grade II or greater) s [29]. Moderate and severe paravalvular leakage was a rare finding confirming the high efficacy of all second-generation devices to prevent this important limitation of TAVI.

## Differentiated THV prosthesis choice

The presented data reflects that TAVI implanters already perform individual decisions for specific patient needs, e.g. more low profile THV use in patients with coronary heart disease and patients with more severe aortic valve calcification or more often use of smaller delivery devices in patients with PAD. The all-comers face-to-face comparison of these four second-generation THVs adds evidence which prosthesis can be chosen, if a lower gradient, less PVL, lower permanent pacemaker rate or less burden in terms of radiation, contrast or pre- and post-dilatation is attempted.

### Study limitation

Due to the retrospective design and the registry-based data acquisition of the study typical limitations apply. To control for confounding baseline variables a propensity score-adjusted analysis was used, but bias due to unknown or unmeasured confounders cannot be excluded, in particular since patients were not randomized to the respective treatment group.

## Conclusions

From our data, we conclude that all currently available THV devices can be safely and effectively used to treat patients with aortic stenosis. There are slight but significant differences in hemodynamics and permanent pacemaker implantation rate between the contemporary second-generation THV devices which imply that patient individual prosthesis selection is necessary to achieve the optimal short- and intermediate-term result. Whether these differences which are mainly due to the prosthesis design and implantation mode translate into significant long-term outcomes or effect prosthesis durability needs to be elucidated.

## Perspectives

### Impact on daily practice

This work demonstrates that despite the high overall clinical efficacy and safety of all investigated THV prostheses patient-based individual THV prostheses selection is necessary to achieve the best clinical result. Further studies are needed to elucidate the best selection criteria. In addition, long-term follow-up studies are needed to evaluate the impact of hemodynamic differences on THV prostheses longevity.

### Supplementary Information

Below is the link to the electronic supplementary material.

Supplementary file1 (DOCX 135 KB)

Supplementary file2 Supplemental Figure 1 Standardized mean differences showing the effect of the propensity score adjustments on the variables included in the propensity score model (age, gender, left ventricular ejection fraction and STS score). (TIF 153 KB)

Supplementary file3 Supplemental Figure 2 NYHA class at baseline (pre, n=24,124) and after 1 year (post, n=6,606) showing a clear improvement following TAVI without any significant difference between the four groups. (TIF 98 KB)

Supplementary file4 (TIF 118 KB)

Supplementary file5 Supplemental Figure 3 Mortality after 30 days (A) and one year (B) with propensity score derived weights adjusting for age, gender, left ventricular ejection fraction and STS score in a subset of patients with aortic annulus between 21 and 27 mm. Mortality was comparable for all studied TAVI prostheses. (TIF 89 KB)
