# Supplementary figures and images for: Comparison of contemporary transcatheter heart valve prostheses: data from the German Aortic Valve Registry (GARY)
Source: Clin Res Cardiol. 2023 Jul 18;113(1):75–85. doi: 10.1007/s00392-023-02242-z (PMC10808310; doi:10.1007/s00392-023-02242-z)

**
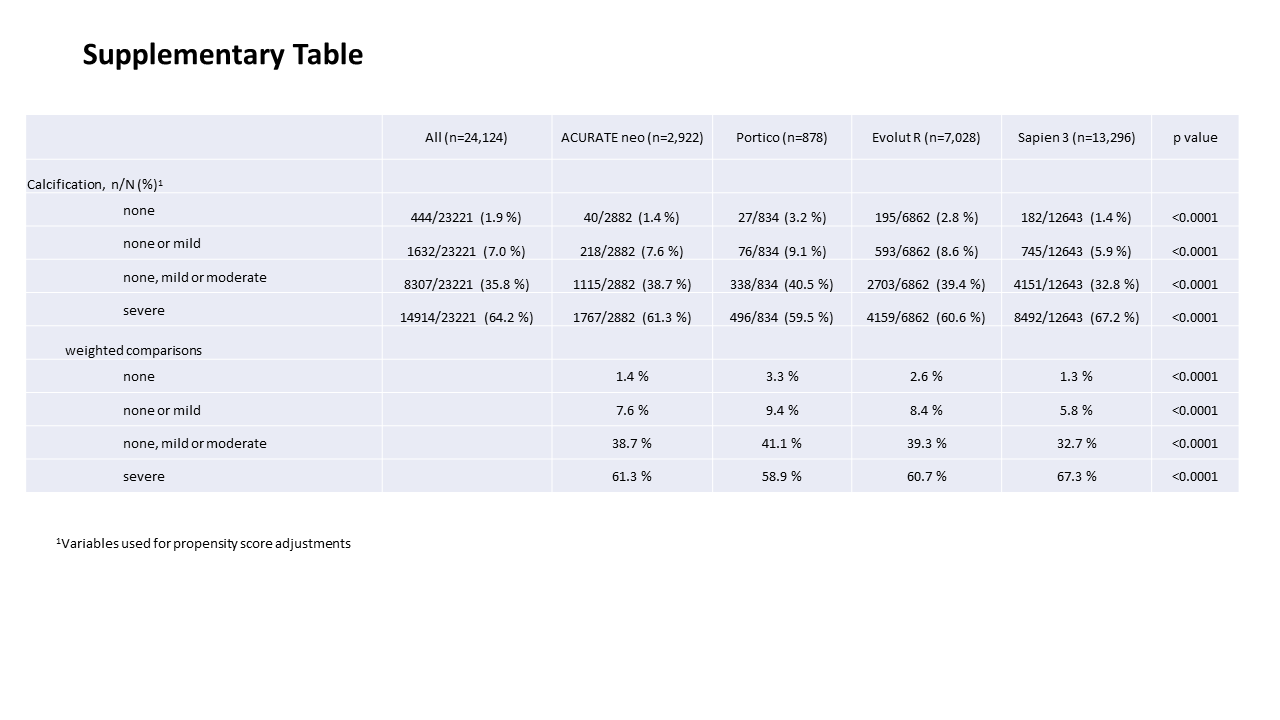
**

Supplement: Supplementary file 1 — Supplementary file1 (DOCX 135 KB) [file 392_2023_2242_MOESM1_ESM.docx]

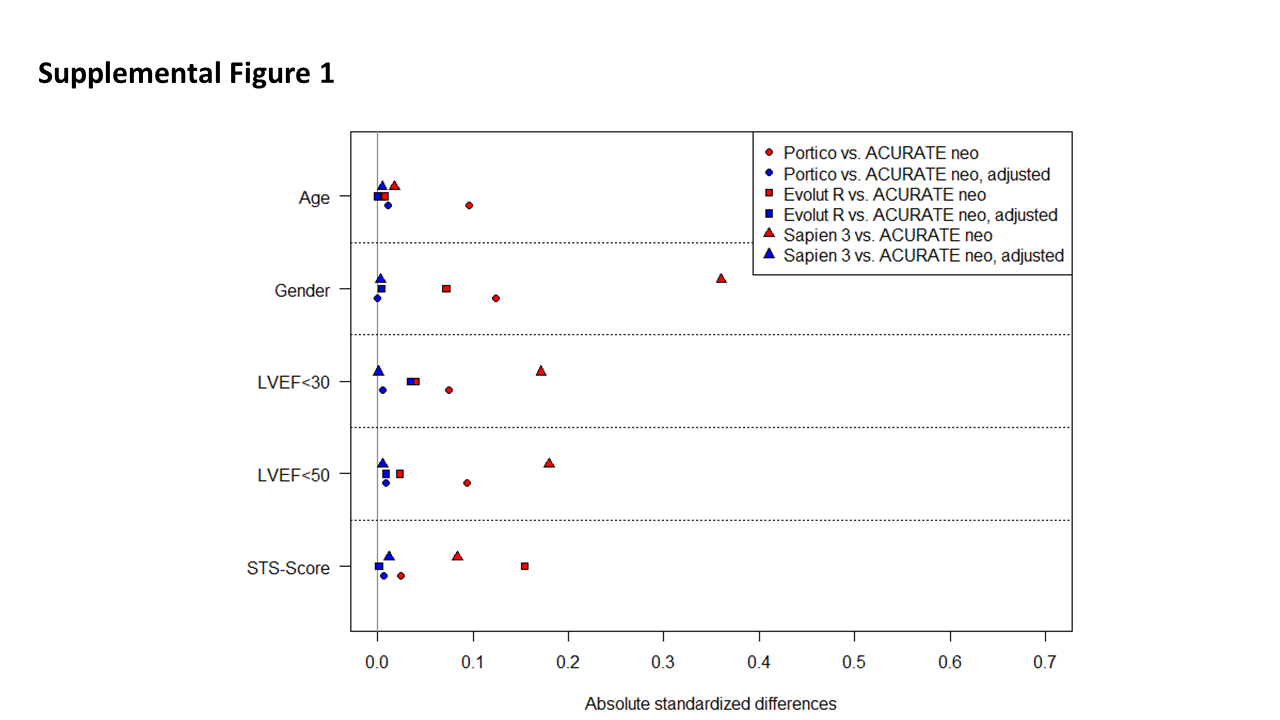

Supplement: Supplementary file 2 — Supplementary file2 Supplemental Figure 1 Standardized mean differences showing the effect of the propensity score adjustments on the variables included in the propensity score model (age, gender, left ventricular ejection fraction and STS score). (TIF 153 KB) [file 392_2023_2242_MOESM2_ESM.tif]

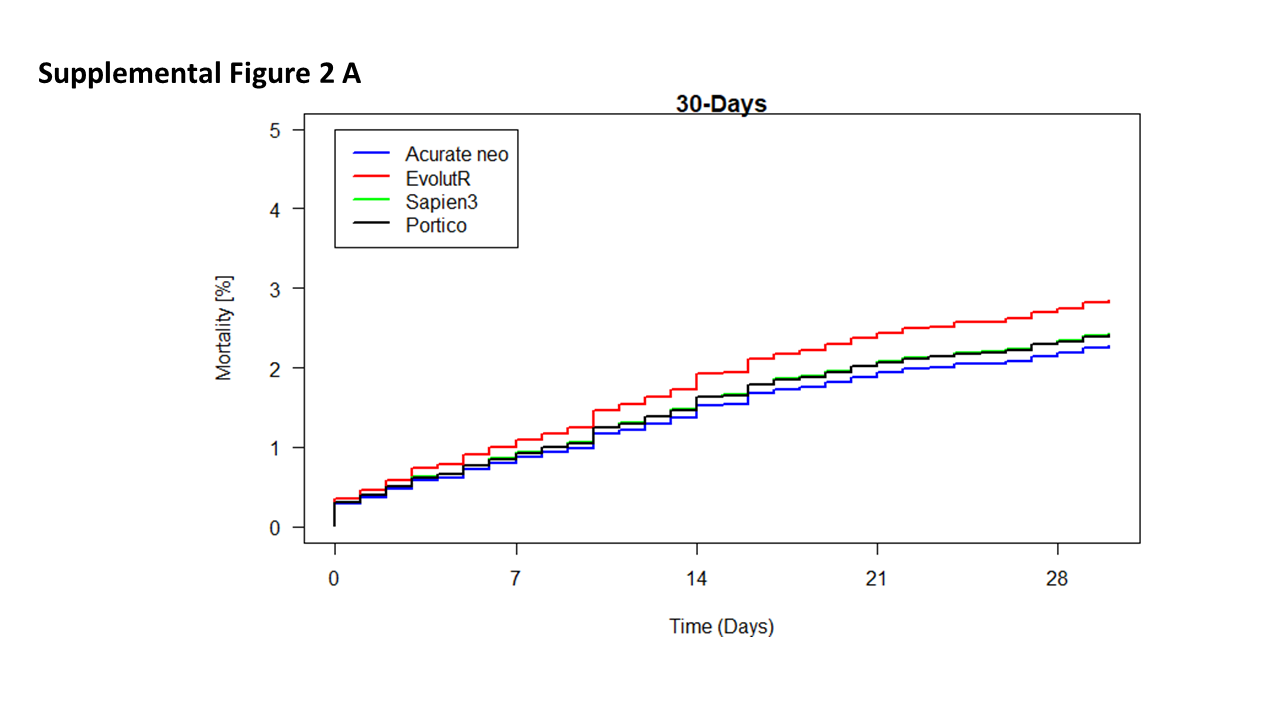

Supplement: Supplementary file 3 — Supplementary file3 Supplemental Figure 2 NYHA class at baseline (pre, n=24,124) and after 1 year (post, n=6,606) showing a clear improvement following TAVI without any significant difference between the four groups. (TIF 98 KB) [file 392_2023_2242_MOESM3_ESM.tif]

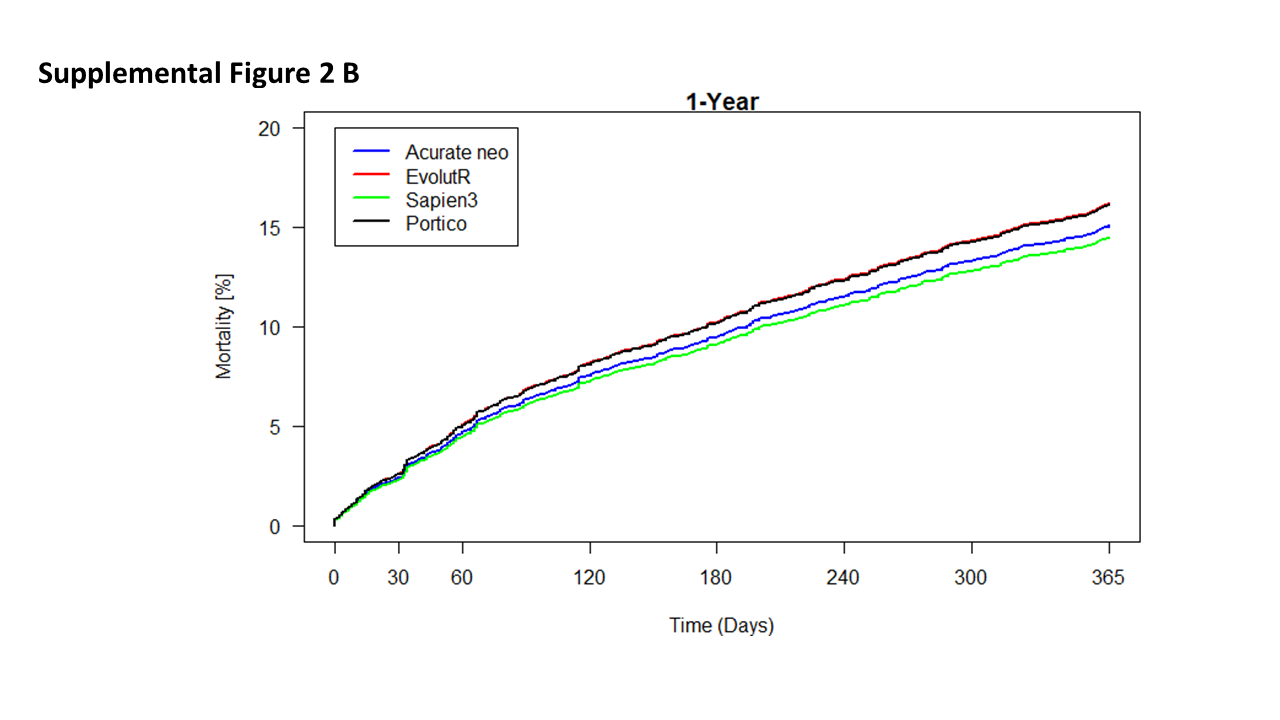

Supplement: Supplementary file 4 — Supplementary file4 (TIF 118 KB) [file 392_2023_2242_MOESM4_ESM.tif]

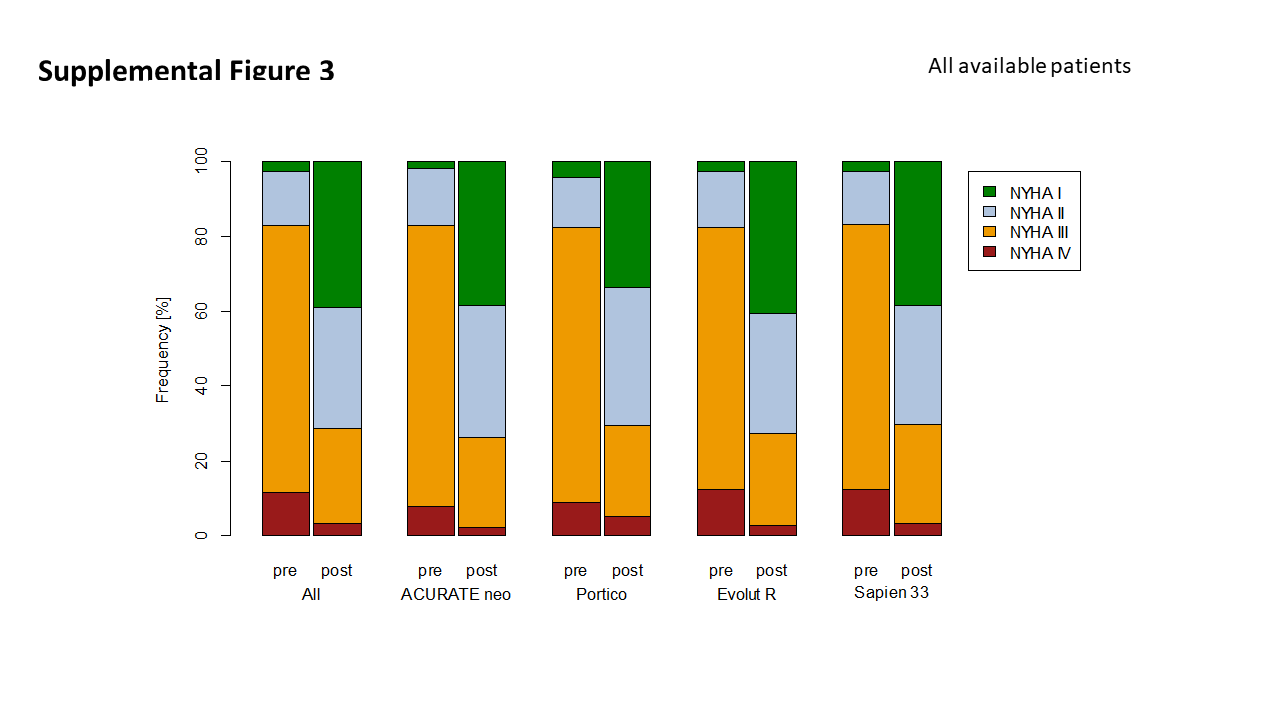

Supplement: Supplementary file 5 — Supplementary file5 Supplemental Figure 3 Mortality after 30 days (A) and one year (B) with propensity score derived weights adjusting for age, gender, left ventricular ejection fraction and STS score in a subset of patients with aortic annulus between 21 and 27 mm. Mortality was comparable for all studied TAVI prostheses. (TIF 89 KB) [file 392_2023_2242_MOESM5_ESM.tif]
